# The role of interactions between bacterial chaperone, aspartate aminotransferase, and viral protein during virus infection in high temperature environment: the interactions between bacterium and virus proteins

**DOI:** 10.1186/1471-2180-13-48

**Published:** 2013-02-26

**Authors:** Yanjiang Chen, Dahai Wei, Yiqian Wang, Xiaobo Zhang

**Affiliations:** 1 Key Laboratory of Conservation Biology for Endangered Wildlife of the Ministry of Education and College of Life Sciences, Zhejiang University, Hangzhou 310058, The People’s Republic of China; 2 Key Laboratory of Marine Biogenetic Resources, Third Institute of Oceanography, State Oceanic Administration, Xiamen 361005, P.R. China; 3 State Key Laboratory for Infectious Disease Prevention and Control, National Institute for Communicable Disease Control and Prevention, Chinese Center for Disease Control and Prevention, Beijing 102206, China

**Keywords:** Protein interaction, Thermophile, Bacteriophage

## Abstract

**Background:**

The life cycle of a bacteriophage has tightly programmed steps to help virus infect its host through the interactions between the bacteriophage and its host proteins. However, bacteriophage–host protein interactions in high temperature environment remain poorly understood. To address this issue, the protein interaction between the thermophilic bacteriophage GVE2 and its host thermophilic *Geobacillus sp.* E263 from a deep-sea hydrothermal vent was characterized.

**Results:**

This investigation showed that the host’s aspartate aminotransferase (AST), chaperone GroEL, and viral capsid protein VP371 formed a linearly interacted complex. The results indicated that the VP371-GroEL-AST complex were up-regulated and co-localized in the GVE2 infection of *Geobacillus sp.* E263.

**Conclusions:**

As reported, the VP371 is a capsid protein of GVE2 and the host AST is essential for the GVE2 infection. Therefore, our study revealed that the phage could use the anti-stress system of its host to protect the virus reproduction in a high-temperature environment for the first time.

## Background

Bacteriophages, like all viruses, rely seriously on their hosts for reproduction [1]. Generally the life cycle of bacteriophage includes seven programmed steps [1,2]. They are phage adsorption on host cell surface, injection of phage genomic DNA into cell, metabolism transition from host to phage, phage genome replication, phage morphogenesis, phage package and lysis of the host [1,2]. Throughout the whole process of phage life cycle, interactions between bacteriophages and host proteins are essential for bacteriophages to set up an efficient infection and to direct the biosynthesis machinery of the host cell toward the reproduction of phages [1-4]. As reported, host RNA polymerase can be a target of phage because most phages use the host’s transcription system in their infection cycles and most interactions take place during the transcription step in the phage infection cycle [1,2,4]. Nevertheless, functions of a number of phage open reading frames (ORFs) driven by strong early promoters remain unknown even in the well-studied bacteriophages T4 and λ [1,4]. Up to date, the mechanisms of most phage–host interactions are still poorly understood [1].

Since thermophilic bacteriophages are more difficult to study, the host–phage interactions in high-temperature environments remain unclear [5]. Because thermophilic bacteria live in high-temperature environments, a powerful machinery to protect against protein denaturation is needed [6]. The use of a molecular chaperone is a well-known strategy for the protection of bacterial proteins. GroEL, one of the most efficient chaperone systems, may be an essential protein for the interactions between thermophilic bacteria and their bacteriophages [5].

GroEL usually has a tetradecameric “cage” structure with seven-fold symmetry that helps fold the nonnative proteins via an ATP-dependent mechanism [7,8]. With the help of the co-chaperonin GroES and ATP, the nonnative protein binds to the apical domain of GroEL and then is encapsulated within the “cage” chamber to finish folding [9,10]. As documented, it was demonstrated that the GroEL can fulfill some essential roles in cells [11-13] and thus is essential for bacterial growth at all temperatures [14,15]. In addition, the GroEL is concerned with the immune responses of host against bacteriophage invasion [7]. In this context, the GroEL system may be involved in the phage infection of the host. To date, there has been plenty of pioneering work on the GroEL system of *Escherichia coli*[7-10,12-15]. However, the function of the GroEL system in the interactions between thermophilic bacteriophages and their hosts remain to be addressed [16].

One of the powerful anti-stress strategies of thermophilic bacteria is the high activity and thermal stability of their enzymes, which can protect their metabolism in high-temperature environments [17]. Aspartate aminotransferase (AST) is a key enzyme involved in the Krebs cycle, which catalyzes the formation of oxaloacetate. AST is also involved in the synthesis of other essential amino acids [18]. AST catalyzes the α-amino group reversible transfer between four- and five-carbon dicarboxylic amino acids and the α-keto-acids by a mechanism named “ping-pong bi-bi”, which is pyridoxal phosphate-dependent [19]. The enzyme plays an important role in the coordination of carbon and nitrogen metabolism in almost all organisms [20]. In prokaryotes, AST represents a central enzyme in the metabolism of Krebs cycle intermediates [21]. ASTs have been classified into the aminotransferase family I and divided into subgroups Ia and Ib. In *Geobacillus*, the enzyme belongs to subgroup Ib. Although our knowledge of AST comes primarily from subgroup Ia, the structures and active site residues of the enzymes in subgroups Ia and Ib are well conserved [22].

In our earlier studies, several thermophilic bacteriophages were isolated from the thermophiles of deep-sea hydrothermal vents [23,24]. Twenty host proteins were found to be involved in the infection of the thermophilic bacteriophage GVE2 [5], a virulent-tailed *Siphoviridae* bacteriophage [25] which infected a thermophilic bacteria *Geobacillus sp.* E263. Our previous study showed that the host’s AST was essential for the GVE2 infection [5]. In the present investigation, the results revealed that a major capsid protein (VP371) of GVE2 and the host AST were interacted with the host GroEL to form a three-protein complex. High temperatures tend to favor protein unfolding and hydrophobic interactions [5]; therefore, it was conceivable that the effect of GroEL was essential in the infection process of thermophilic bacterophages.

## Methods

### Culture of *Geobacillus sp*. E263 and infection of GVE2

The deep-sea thermophile *Geobacillus sp*. E263 (China General Microbiological Culture Collection Center accession no. CGMCC1.7046) was cultured at 60°C with shaking in TTM medium (0.2% NaCl, 0.4% yeast extract, 0.8% tryptone; pH 7.0). The host strain cultures in the mid-exponential phase were infected with its thermophilic bacteriophage GVE2 at a multiplicity of infection (MOI) of 5 and cultured at 60°C.

### Protein recombinant expressions in *E. coli* and antibody preparations

The AST, GroEL and MreB genes of *Geobacillus sp*. E263 and the vp371 gene of GVE2 were cloned into pGEX-4 T-2 vector (Novagen, Germany) and expressed in *E. coli* BL21 (DE3) as glutathione S-transferase (GST)-tagged fusion proteins. The recombinant plasmids were confirmed by DNA sequencing. To obtain the recombinant proteins, the recombinant bacteria were induced using isopropyl-β-D- thiogalactoside (IPTG) when the optical density of bacteria was 0.6 at 600 nm. After further incubation for 12 h at 16°C, the induced cells were harvested by centrifugation at 6,000×*g* for 10 min. The recombinant proteins were purified by affinity chromatography using Glutathione Sepharose resins under native conditions according to the recommended protocol (Qiagen, USA).

The purified recombinant fusion proteins were used as antigens to immunize mice according to a standard procedure [26]. The immunoglobulin G (IgG) fractions of the antiserum were purified with protein A-Sepharose (Bio-Rad) and stored at −80°C until use. As determined by enzyme-linked immunosorbent assay, the antisera dilutions were 1:10,000. The specificity of antibodies was confirmed using Western blotting with the recombinant proteins, virus-infected bacteria and host cells.

### Co-immunoprecipitation (Co-IP)

The GVE2-infected *Geobacillus sp*. E263 was collected by centrifugation at 7,000× *g* for 10 min. The precipitate was re-suspended in 0.1 M Tris–HCl (pH 7.5). After sonication for 5 min, the suspension was centrifuged at 12,000×*g* for 15 min. The appropriate immunoprecipitation antibody was added to the supernatant and incubated for 2 h at 4°C. Protein A Sepharose slurry (Bio-Rad) was subsequently added, followed by incubation for 2 h at 4°C. Nonspecific binding proteins were removed by five successive rinses with phosphate buffered saline (PBS). The Protein A Sepharose was finally eluted with glycine solution (0.1 M; pH 1.8). The eluant was collected and analyzed using sodium dodecyl sulfate-polyacrylamide gel electrophoresis (SDS-PAGE).

### Mass spectrometry (MS) analysis

The protein bands of the SDS-PAGE were excised, trypsinyzed and analyzed using matrix-assisted laser desorption ionization-time-of-flight (MALDI-TOF) MS. A 1.5-μL aliquot was spotted onto a MALDI-TOF sample plate with an equal volume of matrix, a saturated solution of α-cyano-4-hydroxycinnamic acid (Sigma, USA) in 0.1% trifluoroacetic acid and 50% acrylonitrile. The samples were analyzed using a Bruker AutoFlex MALDI-TOF mass spectrometer (Bruker Daltonics, USA). All peptide mass finger printings were externally calibrated using standard peptide mixtures and internally calibrated using the masses of trypsin autolysis products to reach a typical mass measurement accuracy of 100 ppm. All acquired sample spectra were processed using Bruker Flexcontrol 2.4 operation software (Bruker Daltonics) in a default mode with an MS tolerance of 0.2 Da and a tandem MS tolerance of 0.6 Da. Protein identification was performed using Mascot software (version 2.1; Matrix Science, London, UK) and GPS Explorer software (version 3.6; Applied Biosystems, USA) against the NCBInr database and the ORF database of *Geobacillus kaustophilus* HTA426 in a local database that was generated using a shotgun approach. To eliminate protein redundancy in the database under different names and accession numbers, the single protein member belonging to the species *G. kaustophilus* HTA426 or otherwise had the highest protein score (top rank) was singled out from the multi-protein family.

### Northern blot analysis

Total RNAs were respectively isolated from thermophilic *Geobacillus sp*. E263 before and after GVE2 infection using Trizol reagent (Invitrogen, USA), followed by incubation with RNase-free DNase I (TakaRa, Japan) for 30 min at 37°C. After electrophoresis on a 1.2% agarose gel in 1× Tris-borate-ethylenediaminetetraacetic acid buffer, the RNAs were transferred to a nylon membrane (Amersham Biosciences, USA). The blots were probed with digoxigenin (DIG)-labeled vp371, GroEL, or AST, respectively. Bacterial 16S rRNA gene was used as a control. In vitro RNA labeling, hybridization, and signal detection were conducted according to the manufacturer's instructions for the DIG High Prime DNA Labeling and Detection Starter Kit II (Roche, Germany).

### Western blot

Protein samples separated by SDS-PAGE were transferred to a nitrocellulose membrane (Bio-Rad) in electroblotting buffer (25 mM Tris, 190 mM glycine, 20% methanol; pH 8.5) for 70 min. The resulting membrane was immersed in blocking buffer (0.1% skim milk, PBS; pH 7.2) at 4°C overnight, followed by incubation with a polyclonal mouse anti-GST-AST IgG, anti-GST-GroEL IgG or anti-GST-VP371 for 3 h, respectively. The membrane was then incubated in alkaline phosphate-conjugated goat anti-mouse IgG (Sigma) for 1 h and detected using NBT and BCIP solutions (BBI, Canada).

### Glutathione S-transferase (GST) pull-down assay

The purified GST, GST-MreB, GST-AST and GST-VP371 proteins were incubated with glutathione beads for 2 h at 4°C. The overnight cultures of *Geobacillus sp.* E263 and Δast mutant were collected by centrifugation at 7000×g for 30 min and resuspended with GST binding buffer [200 mM NaCl, 20 mM Tris–HCl, 1 mM EDTA (ethylene diamine tetraacetic acid), 1 mM PMSF (phenylmethanesulfonyl fluoride), pH 7.6]. The suspension was sonicated for 15 min and centrifuged at 10000×g for 15 min. Subsequently the supernatant was incubated with GST, GST-MreB, GST-AST or GST-VP371 coupled glutathione beads for 5 h at 4°C with gentle rotation. Non-specific binding proteins were removed by five washes using GST binding buffer. Then the proteins bound were eluted with elution buffer (10 mM glutathione, 50 mM Tris–HCl, pH 8.0), and detected by Western blot.

### Bacterial two-hybrid assay

To characterize the interactions between AST and GroEL of *Geobacillus sp*. E263 and the VP371 of GVE2, bacterial two hybrid assay was conducted, using the BacterioMatch two-hybrid system (Stratagene, USA). This system uses a reporter gene cassette that is incorporated into an F’ episome and contains the ampicillin (carbenicillin resistance) and β-galactosidase genes. The reporter strain (kanamycin resistance) harbors lacIq on the F’ episome to repress bait and target synthesis. If the bait (on the pBT vector, which has chloramphenicol-resistance) and target (on the pTRG vector, which has tetracycline resistance) fusion proteins interact with each other, transcription of the reporter genes are activated and represent carbenicillin resistance. Screening for protein–protein interactions involves assaying for growth on LB agar with chloramphenicol, tetracycline, carbenicillin and kanamycin (LB-CTCK). The AST gene was amplified using primers 5^′^-GT*GCGGCCGC*ATGAAGCTGGCAA AACGG-3^′^ (NotI in italics) and 5^′^-GT*GGATCC*TTAGGCCCGCGCCTCCAT-3^′^ (BamHI in italics) and cloned into the pBT (Stratagene, USA) to construct the pBT-AST plasmid. The GroEL gene was cloned into the pTRG (Stratagene) using primers 5^′^-AT *GCGGCCGC*ATGGCAAAACAAATCAAG-3^′^ (Not I in italics) and 5^′^-AT*CTCGAG*T TACATCATGCCGCCCAT-3^′^ (XhoI in italics), yielding the pTRG-GroEL plasmid. To construct the recombinant pBT-vp371, the vp371 gene was cloned into the pBT with primers 5^′^-GT*GCGGCCGC*ATGCCGAAGGAATTACGTG AAC-3^′^ (NotI in italics) and 5^′^-GT*GGATCC*TTAAGCAAGTTGTACTTCACCG-3^′^ (BamHI in italics). For the pTRG-vp371 construct, the vp371 gene was cloned into the pTRG with primers 5^′^-AT*GCGGCCGC*ATGCCGAAGGAATTACGTGAAC-3^′^ (NotI in italics) and 5^′^-AT*CTCGAG*TTAAGCAAGTTGTACTTCACCG-3^′^ (XhoI in italics). All of the recombinant plasmids were confirmed using DNA sequencing.

The constructs of pBT and pTRG were co-transformed into the competent cells of the BacterioMatch® Two-Hybrid System Reporter Strain (Stratagene). The resulting bacterial cells were subsequently plated on LB medium containing tetracycline, chloramphenicol, and kanamycin or the LB-CTCK medium. The plates were incubated for 24–36 h at 30°C and then the colonies were examined.

### Antibody labeling

The antibodies against AST, GroEL, and VP371 were respectively labeled using an Alexa Fluor®532 Protein Labeling Kit, 350 Protein Labeling Kit, and 488 Protein Labeling Kit according to the manufacturer’s instructions (Invitrogen). As controls, the antibodies against GST and MreB were labeled with Alexa Fluor® 488 Protein Labeling Kit, respectively. Briefly, the antibody solution was added to1 M bicarbonate (pH 8.3) and then mixed with the reactive dye. After incubation at room temperature for 1 h, the mixture was loaded onto the purification resin. PBS (pH 7.4) was subsequently added and the labeled antibody was collected.

### Immunofluorescence microscopy

Overnight cultures of *Geobacillus sp*. E263 were diluted in TTM medium containing 0.01 M MgCl_2_ and grown at 60°C. When the OD_600_ reached 0.3–0.6, the bacteria were infected with GVE2 at an MOI of 5. For imaging, the GVE2-infected and virus-free *Geobacillus sp*. E263 were immobilized on slides (Sigma) covered with a thin 1% agarose film. The labeled antibodies against AST, GroEL, VP371, GST, and/or GroEL were added to the cultures that were permeabilized by 0.1% Triton X-100. The mixtures were incubated overnight at 4°C. The samples were examined under a Leica TCS SP5 confocal microscope (Germany). The digital images were acquired and analyzed using LAS AF version 2.0.0 software. Images of fluorescent samples were deconvolved within LAS AF and assembled using Adobe Photoshop version 7. Image manipulation was kept to a minimum.

### Isothermal titration calorimetry

All proteins were purified and dialyzed into PBS (pH7.4) overnight at 4°C. Protein concentration was determined using ultraviolet absorbance at 280 nm on a NanoDrop ND-1000 spectrophotometer (NanoDrop Technologies, Wilmington, DE, USA). The titration experiments were conducted on a VP-ITC isothermal titration calorimeter (ITC) from MicroCal™, Inc. (Northampton, MA, USA) at 25°C. A 250-μL syringe was used for the ITC injections at a stirring speed of 307 rpm. The injections (10 μL each) were administered every 120 s. The AST, VP371, and GST concentrations in the syringe were 30–50 μM, whereas the GroEL, AST, and GST concentrations in the cell were 6–10 μM. All samples were degassed for 10–30 min prior to use, and all experiments were done at least in triplicate. To calculate the thermodynamic changes of the interactions between GroEL and the other two proteins, the interactions were measured at 35°C, 50°C, and 60°C. The results were analyzed using Origin 7(MicroCal™ LLC ITC) and fitted to a “three sets of sites” model. In this way, the thermodynamic association constant (Ka) and enthalpy change (ΔH) can be calculated directly. The Gibbs free energy change (ΔG) was calculated using the equation ΔG =−RTlnKa, where R was the molar gas constant and T was the absolute temperature at which the experiment was conducted. The entropy change of the interaction was calculated according to the equation TΔS = ΔH − ΔG.

## Results

### The interactions between the bacterial chaperone GroEL, AST, and the viral VP371 proteins

**Figure 1:**
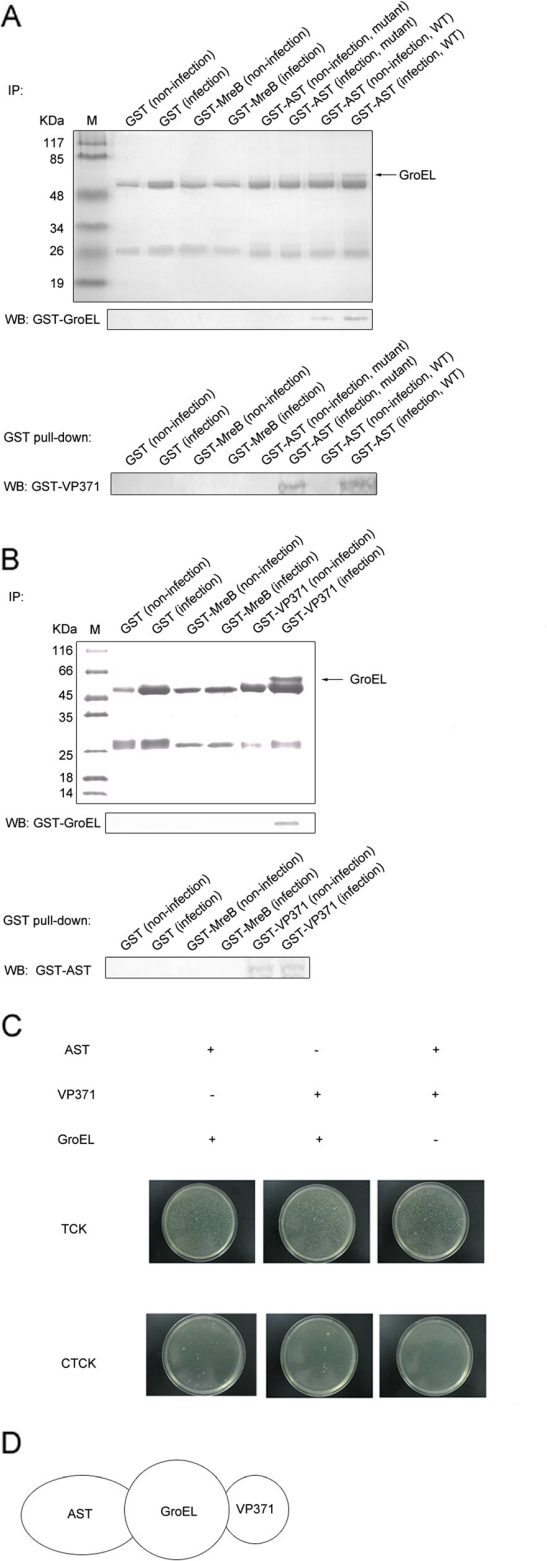
**Interactions among the bacterial GroEL, aspartate aminotransferase (AST), and viral VP371 proteins. (A)** Interaction between AST and GroEL. The cultures of GVE2-infected or non-infected thermophilic *Geobacillus* sp. E263 (wild-type, WT) were used for co-immunoprecipitation (Co-IP) with antibodies against GST, GST-MreB or GST-AST and used for GST pull down with GST, GST-MreB or GST-AST. The mutant of AST (∆ ast) was also included in the Co-IP assays. The antibodies used for IP were indicated at the top. The resulting Co-IP solutions were subsequently subjected to sodium dodecyl sulfate- polyacrylamide gel electrophoresis (SDS-PAGE; Coomassie staining) (up) and Western blot (down), respectively. The proteins used for GST pull down were presented at the top. For Western blot, the antibodies used were shown on the left. The arrow showed the protein identified using mass spectrometry. M, protein marker. **(B)** Interaction between VP371 and GroEL. The cultures of GVE2-infected or non-infected thermophilic *Geobacillus* sp. E263 were used for Co-IP with the VP371-specific, GST-MreB-specific or GST-specific antibodies and used for GST pull down. The resultant Co-IP solutions were subjected to SDS-PAGE (up) and Western blot (down), respectively. The mass spectrometric identification of protein was shown with an arrow. The proteins used for GST pull down were indicated at the top. M, protein marker. **(C)** Bacterial two-hybrid analysis of interactions among GroEL, aspartate aminotransferase and VP371 proteins. *E. coli* cells were co-transfected with recombinant plasmids as indicated at the top. The transformants were grown in agar plates containing the selective antibiotics TCK (tetracycline+chloramphenicol+ kanamycin) or CTCK (carbenicillin+tetracycline+ chloramphenicol+kanamycin). **(D)** Model of the linear interactions in the GroEL-aspartate aminotransferase-VP371 complex.

In our earlier study [5], we found that bacterial AST was required for phage GVE2 infection. To reveal the proteins that interacted with AST, the Co-IP assay was conducted using the antibody against AST. The results showed that a protein was specifically bound to AST (Figure 1A), while no protein was bound to an unrelated fusion protein control GST-MreB or GST in conditions of non-infection or infection with GVE2 (Figure 1A). When the AST mutant was used in the Co-IP assays with AST antibody, no protein bound to AST was found (Figure 1A). As identified by MS, the protein bound to AST was chaperone GroEL of *Geobacillus* sp. E263. The mass spectrometric result was confirmed using Western blot analysis (Figure 1A). These data revealed the existence of an interaction between AST and GroEL of *Geobacillus* sp. E263.

When the viral major capsid protein VP371 of GVE2 was investigated with Co-IP, the VP371 was specifically bound to a protein that was identified to be the bacterial GroEL using MS (Figure 1B). In the controls, no protein was bound to GST or GST-MreB. The interaction between viral VP371 and host GroEL proteins was confirmed using Western blotting (Figure 1B). The GST pull-down results showed that the viral VP371 protein and the host AST protein was interacted with the host GroEL protein (Figure 1A and 1B), suggesting the existence of the VP371-GroEL-AST complex.

To reveal the interactions in the VP371-GroEL-AST complex, the bacterial two-hybrid system was conducted. Only proteins that interacted with each other could induce growth of the reporter strain in LB-CTCK medium (Figure 1C). The results presented that protein–protein interactions existed between VP371 and GroEL and GroEL and AST, but not between VP371 and AST (Figure 1C). Thus, we proposed that these three proteins were linearly bound to each other in the VP371-GroEL-AST complex in high temperature environment (Figure 1D).

### Expression profiles of host AST, GroEL, and viral vp371 genes in vivo

**Figure 2:**
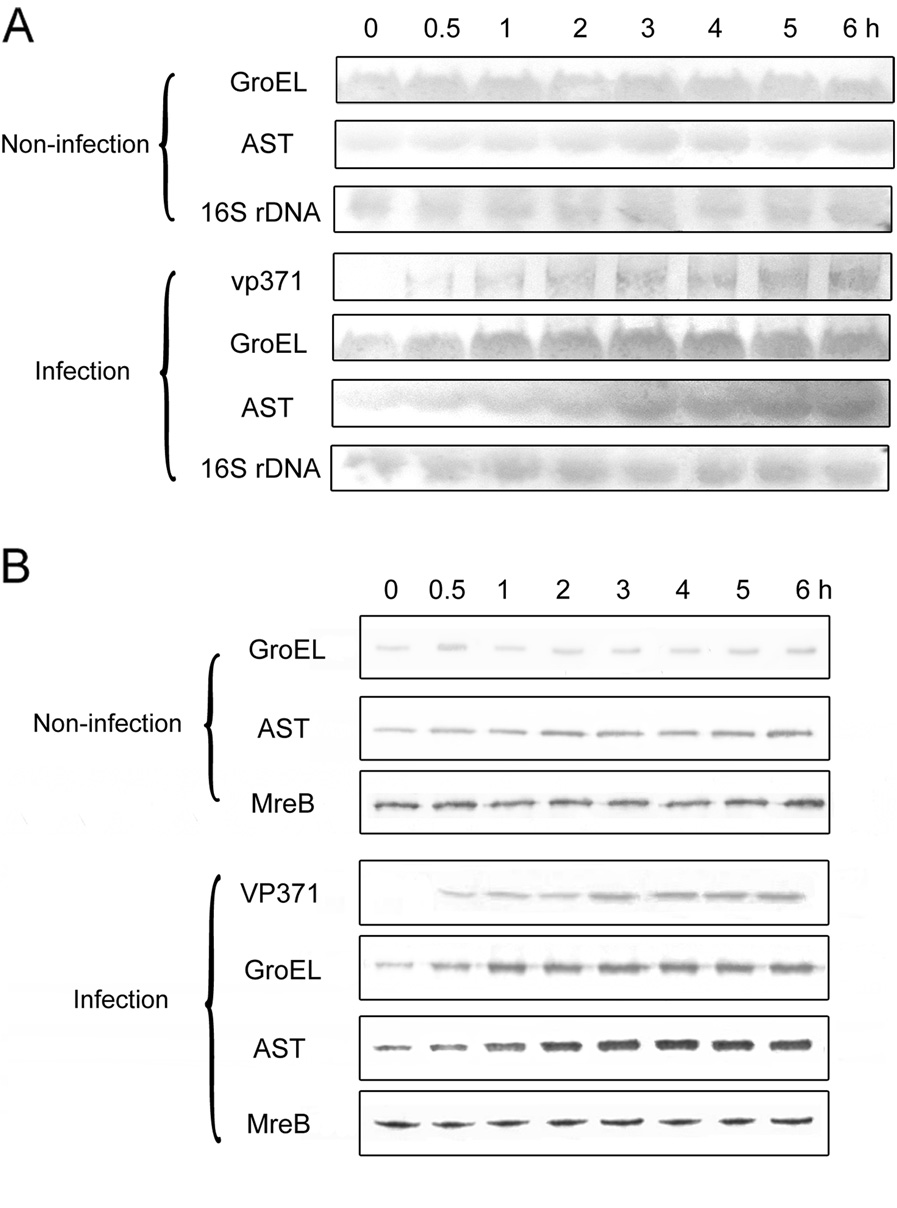
**Expression profiles of host aspartate aminotransferase, GroEL, and viral vp371 genes in GVE2-infected and non-infected *Geobacillus *sp. E263.** The *Geobacillus* sp. E263 was challenged with GVE2. At various times post-infection (p.i.), the GVE2-infected and non-infected bacteria were characterized using Northern blots with gene-specific probes **(A)** and Western blots with protein-specific antibodies **(B)**, respectively. The probes and antibodies were indicated on the left side. The bacterial 16S rDNA and MreB were used as controls. The lane headings showed the time post-infection in hours.

To characterize the expression profiles of the host AST, GroEL, and viral VP371 in response to bacteriophage challenge in high temperature environment, *Geobacillus* sp. E263 was infected with GVE2 followed by Northern and Western blots. The results showed that the AST, GroEL and vp371 gene transcriptions were up-regulated after GVE2 infection by comparison with the non-infected bacteria (Figure 2A). The Western blots yielded similar results to those of Northern blot analyses (Figure 2B). These results indicated that the thermophilic host AST, GroEL, and viral VP371 proteins were involved in the GVE2 infection to its host in high temperature environment.

### Co-localization of host AST, GroEL and viral VP371 proteins during bacteriophage infection

**Figure 3:**
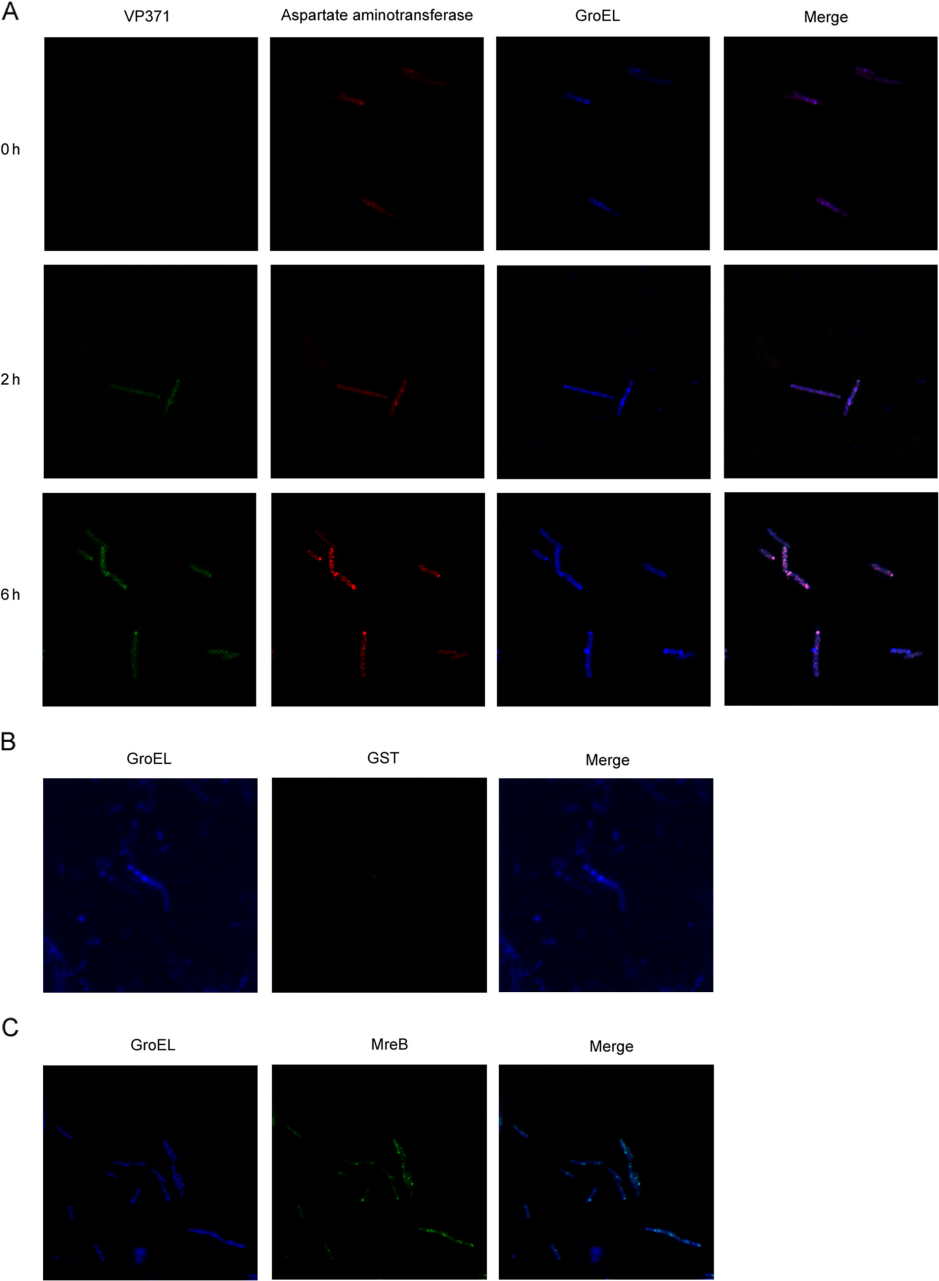
**Co-localization of host aspartate aminotransferase (AST), GroEL, and viral VP371 in *Geobacillus *sp. E263.** The host bacteria were challenged with GVE2. At different time post-infection, the GVE2-infected *Geobacillus* sp. E263 was labeled with the antibodies against the AST, GroEL, or VP371 **(A)**. The GST **(B)** and the GST-MreB **(C)** were used as controls to detect the nonspecific co-localization with GroEL at 2 h post-infection. The bacteria were examined under a fluorescence microscope. The lane headings indicated the labeled proteins. The numbers showed the time post-infection in hours.

To characterize the VP371-GroEL-AST interactions during GVE2 infection, these three proteins were labeled and examined using immunofluorescence microscopy. The results indicated that the host AST, GroEL, and viral VP371 proteins were co-localized in the GVE2-infected *Geobacillus* sp. E263 (Figure 3A). In the virus-free *Geobacillus* sp. E263, however, the AST and GroEL were bound to each other (Figure 3A), while no signal was observed in the GST control and no obvious co-localization was found between the GST-MreB control and GroEL proteins (Figures 3B and 3C). Considering the importance of the VP317 and AST proteins in the GVE2 infection [5,25], the immunofluorescence microscopy results suggested that the VP371- GroEL-AST complex might be involved in the bacteriophage infection in high temperature environment.

### Thermodynamic characterization of the VP371-GroEL-AST interactions

**Figure 4:**
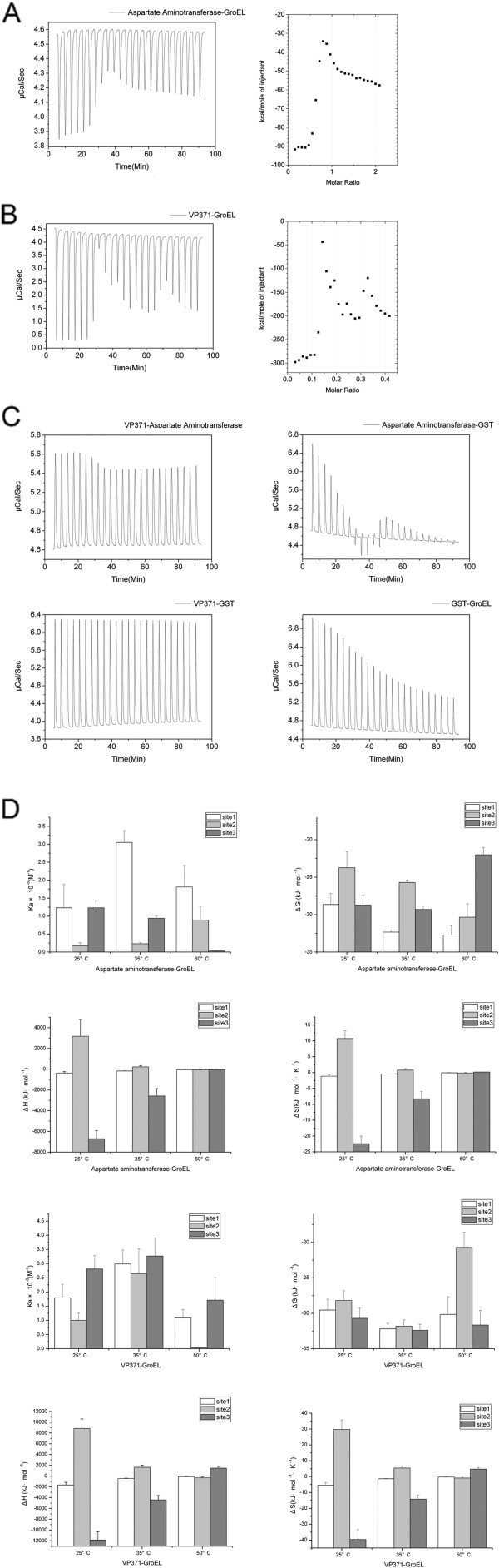
**Thermodynamic characterization of the VP371-GroEL-aspartate aminotransferase (AST) interactions.** The purified proteins of VP371-GroEL-AST linear complex and GST as control group were combined for isothermal titration calorimetry measurements. The experiment was performed at 25°C in phosphate buffered saline (pH 7.4) with 10-μL injections. **(A)** Thermogram (left) and binding isotherm (right) for the interaction between AST and GroEL. Concentrations of AST and GroEL were 44.5 and 8.5 μM, respectively. **(B)** Thermogram (left) and binding isotherm (right) for the interaction between VP371 and GroEL. Concentrations of VP371 and GroEL were 38.5 and 6.5 μM, respectively. **(C)** Thermogram for the titrations of 38.5 μM VP371 to 7 μM AST, 44.5 μM AST to 8.5 μM GST, 38.5 μM VP371 to 6.5 μM GST, and 44.5 μM GST to8.5 μM GroEL. **(D)** Thermodynamic parameters for binding of aspartate aminotransferase-GroEL and VP371-GroEL at different temperatures. All experiments were performed in phosphate buffered saline (pH 7.4) using isothermal titration calorimetry. Site 1–3, three different sites of the simulated interaction.

The binding properties of the interactions in the VP371-GroEL-AST linear complex were characterized by ITC. Figure 4 showed a thermogram for all 3 kinds of protein–protein combinations and binding isotherms only for the valuable interaction (AST-GroEL or VP371-GroEL).

An obvious difference in the binding properties between the valuable interactions and the control combinations (AST-VP371, AST-GST, VP371-GST and GST-GroEL) was generally observed. The isotherm for the binding of AST to GroEL (Figure 4A) and VP371 to GroEL (Figure 4B) released endothermic heat, which could be best fitted to the “three sets of sites” binding model in the Origin software, whereas the control combinations released exothermic heat (Figure 4C, except for AST-GST group but also mainly exothermic heat) and no binding was detected. This analysis suggested three kinds of binding interactions between GroEL and AST or VP371. To evaluate the interactions between VP371, GroEL and AST at different temperatures, the thermodynamic parameters were measured at 25°C, 35°C, 50°C or 60°C. The thermogram results showed that the VP371 and GroEL, and GroEL and AST proteins were interacted (Figure 4D). Because ITC assay, a temperature sensitive experiment, might not keep a stable environment at high temperature. When the temperature reached at or over 50°C, the thermodynamic parameters became unstable (Figure 4D).

## Discussion

Bacteriophages are known significant genetic regulators with a remarkable ability to modify a host’s biomachinery including DNA replication or transcription or RNA translation [7,27]. Although plenty of bacteriophages have been extensively studied, thermophilic bacteriophages and bacteriophage–host interactions remain poorly understood. Thermophilic phages in mud pots, solfataric fields, hot springs, and deep-sea hydrothermal vents are undoubtedly very important in the genetic diversity, microbial mortality, and nutrient cycling of these extreme environments [23,28-31]. Thus, biochemical and genetic studies on the relationship between thermophilic phages and their hosts will reveal new insights in the life within the extreme biosphere. In the present study, the interaction between the bacteriophage GVE2 and its host thermophilic *Geobacillus sp*. E263 from a deep-sea hydrothermal field was characterized. We found that the host AST, GroEL, and viral VP371 proteins formed a linearly interacted complex.

The ITC results provided a thermodynamic characterization of the complex interactions. First, the endothermic thermograms showed a similar binding mode for GroEL to AST and VP371 (Figures 4A and 4B), and the ITC peak suggested an exothermic progress caused by the depolymerization of the known polymers GroEL and VP371. However, the details of their interactions were much more complicated because they were not fitted to simple models. The thermodynamic parameters provided more information about the interactions (Figure 4D). The ΔH value was the heat associated with the making and breaking of non-covalent bonds from the free to the bound state. The ΔS value indicated on the total change in the degrees of freedom [32-35]. In this study, although the results derived at 50 and 60°C were unstable in the high-temperature environment, the affinities (represented by ΔG or K) of interactions at 35°C remained higher than those at 25°C, and this instability in high temperature environment suggested that the host and the phage require a mechanism for protecting their proteins and surviving the heat.

The AST can catalyze the amino group transfer between amino acids and the 2-oxo acids, which plays a central role in amino acid metabolism from bacteria to mammals [36]. Our earlier studies revealed that AST is required for the GVE2 infection and that the VP371 is a capsid protein of GVE2 [5,25]. As evidenced, the chaperone GroEL provides assistance with the folding of nonnative proteins to their native states [9]. In this context, the host GroEL might play very important roles in bacteriophage infection in high temperature environment through facilitating the correct folding of the host AST and the viral capsid protein VP371. In our study, it was found that the knockout of *Geobacillus* sp. E263 GroEL led to the lethality of bacterium (data not shown). To reveal the roles of the AST-GroEL-VP371 interactions in bacteriophage infection, the function of GroEL merited to be further investigated in future.

The GroEL, which is well investigated in *E.coli*, can provide assistance to the folding of proteins in an adenosine triphosphate (ATP)-dependent manner [7,8]. With the help of a co-chaperonin GroES and ATP, the nonnative protein binds to the apical domain of GroEL and is then encapsulated within the “cage” chamber to finish its folding [9,10]. As reported, GroEL is essential for the growth of bacteria at all temperatures [14,15]. The GroEL/GroES machine is concerned with the defense strategies of hosts against their bacteriophages [7]. Therefore, the GroEL may be involved in bacteriophage infections. To date, the only case about the interaction between the GroEL and bacteriophage comes from bacteriophage T4. Bacteriophage T4 expresses Gp31, a protein that is uniquely essential for the correct maturation of Gp23, the major T4 capsid protein. The Gp31 protein can substitute for GroES in *E. coli* to facilitate the bacteriophage infection. In the GroEL/GroES system, Gp31 rather than GroES can ensure the proper folding of Gp23 for unknown reasons [37]. The sequence analysis in our study showed that no homologous protein of Gp31 in the deduced open reading frames (ORFs) of GVE2. The direct interaction between the host GroEL and the viral VP371 protein, therefore, was related to the host GroEL system, which was used by the bacteriophage GVE2 to ensure viral protein synthesis in high temperature environment. The present investigation on thermophilic GroEL provided a clue to understanding the host–virus interaction in the deep-sea vent ecosystems.

## Conclusions

This context revealed the AST-GroEL-VP371 linear complex which was up-regulated in the infection of GVE2. It could be inferred that the interaction between the host GroEL and the viral VP371 proteins facilitated the synthesis of the major capsid protein VP371 in the GVE2 bacteriophage life cycle. In addition, GroEL in the host cells could facilitate the correct folding of host AST, which provided more effective amino acid metabolism to ensure the protein synthesis of bacteriophages in high temperature environment.

## Competing interests

The authors declare that they have no competing interests.

## Authors’ contributions

Yanjiang Chen and Xiaobo Zhang conceived the experimental design and wrote the manuscript. Dahai Wei conducted the Co-IP, Western blot, Northern blot and bacterial two-hybrid assays of AST and GroEL. Yiqian Wang performed the interaction between VP371 and GroEL. Yanjiang Chen carried out the immunofluorescence microscopy and isothermal titration calorimetry experiments and analyzed the data. All authors have read and approved the final version of the manuscript.
